# The use of anterior segment optical coherence tomography (ASOCT) in demonstrating recurrence of vitreoretinal lymphoma (VRL) in the anterior vitreous

**DOI:** 10.1186/s40942-019-0169-8

**Published:** 2019-08-20

**Authors:** Vlad Diaconita, Heba Rihani, Virginia Mares, Marcio B. Nehemy, Sophie J. Bakri, Jose S. Pulido

**Affiliations:** 1 Department of Ophthalmology, Ivey Eye Institute, Western University, London, ON Canada; 2 Department of Ophthalmology, Mayo Clinic, 200 First Street, SW, Rochester, MN 55905 USA; 3 Department of Ophthalmology, Belo Horizonte, Brazil

**Keywords:** Primary vitreoretinal lymphoma, Vitreoretinal lymphoma, CNS lymphoma, Anterior segment OCT, ASOCT, VRL

## Abstract

**Background:**

Primary vitreoretinal lymphoma (VRL) is a rare disease with 30–380 new cases in the United States per year. Its insidious process and spread to the central nervous system (CNS) leads to a mean 5-year survival rate from 41.4 to 71%. Medical treatment of VRL has been summarized extensively in the literature and involves intraocular rituximab and methotrexate as first line agents in unilateral VRL, with systemic chemotherapy to be considered in bilateral or CNS-involving disease. In addition, therapeutic “debulking” vitrectomy has been reported in the literature, with some limited success. Despite this, recurrence rate is high and should always be suspected in the setting of new inflammation. Anterior segment optical coherence tomography (ASOCT) has not been previously used to image VRL recurrence in the anterior vitreous.

**Case presentation:**

A 63-year-old man, with VRL was found to have cells and debris in the anterior vitreous, 10 months after his first vitrectomy, intravitreal rituximab and methotrexate. Since the patient was phakic at the time of initial vitrectomy, the anterior vitreous had not been removed. ASOCT confirmed the findings. Subsequent surgery removed the lens and debris. Both the patient’s vision and ASOCT improved.

**Conclusions:**

We suggest that ASOCT of the anterior segment is a useful diagnostic tool to monitor for recurrence of VRL. In biopsy-proven VRL, phakic patients who undergo therapeutic vitrectomy, should also be considered for lens extraction and anterior vitrectomy to limit recurrences.

## Background

Primary vitreoretinal lymphoma (VRL) is a rare disease with approximately 30-380 new cases in the United States per year [1–4]. Of these cases, 95% are CD20+, diffuse large B-cell lymphomas (DLBCL), with a minority being of the T-cell variety [5–9]. It is most commonly diagnosed in the 7th decade of life and presents with an increase in floaters and a painless decrease in vision. Its insidious process and spread to the central nervous system (CNS) leads to a mean 5-year survival rate from 41.4 to 71% [7, 10–15]. Poor outcomes can be attributed to delay in diagnosis and treatment [4, 7, 10, 16, 17].

Elevated IL-10/IL-6 ratios [18, 19], and more recently polymerase chain reaction (PCR) for monoclonality and MYD88 genetic testing [8, 11, 20–23] are reliable and objective means of diagnosis and follow-up for disease recurrence. However, they require vitreous samples or a therapeutic vitrectomy [2, 10]. Medical treatment of VRL has been recently summarized extensively in the literature, and involves intraocular rituximab and methotrexate as first line agents in unilateral VRL, with systemic chemotherapy to be considered in bilateral or CNS-involving disease [4, 9, 24–32]. In addition, therapeutic “debulking” vitrectomy has been reported in the literature, with some limited success [33, 34]. Despite this, recurrence rate is high and should always be suspected in the setting of new inflammation [14, 17].

We present an interesting case of a 63-year-old male with a history of diagnostic vitrectomy, and anterior vitreous recurrence of VRL, which was well-visualized with ASOCT.

## Case presentation

A 63-year-old male was referred to Mayo Clinic for CNS symptoms of left-sided arm weakness and leg weakness, which started 6 months prior. Magnetic Resonance Imaging (MRI) of the brain with contrast revealed a homogeneously enhancing right, frontal lobe mass, which was biopsy positive for DLBCL with MYD88 L265P alteration detected by PCR. He subsequently completed 4 cycles of high dose systemic methotrexate, rituximab and temozolomide. Otherwise, he had no previous ocular history or ocular medications. He had a medical history of benign prostatic hypertrophy and deep venous thrombosis thought to be secondary to DLBCL.

By the time he presented to us, he described a 2-year history of floaters and haze in the right eye. According to the patient, testing had been performed in the community to rule out VRL, which was negative. The authors did not have access to detailed results of these investigations. The reason for referral had been for an increase in floaters in the right eye and new onset of similar symptoms in the left eye. There was no associated pain, redness or photophobia.

On examination, his vision was count fingers (CF) in the right eye and 20/25—in the left eye. The intraocular pressure (IOP) was 12 mmHg in each eye. On slit lamp examination, he was found to be phakic, with 3+ cells in the anterior vitreous of both eyes. Bilaterally, there were 0.5+ cells (high power field) in the anterior chamber and trace flare as well as fine keratic precipitates inferiorly on the endothelium. Posterior segment dilated examination revealed a hazy vitreous with no view in the right eye, and a slight haze in the left eye. There were no clinically visible lesions in the choroid or retina of the left eye. B-scan ultrasound confirmed the pathology was limited to the vitreous. MRI of the brain did not show new lesions. Given his clinical presentation and history, a presumed diagnosis of VRL was made and the patient was consented for a pars plana vitrectomy of the right eye with intraoperative injection of 400 mcg/0.1 cc of methotrexate. The left eye was treated medically with alternating rituximab 1 mg/0.1 cc and methotrexate 400 mcg/0.1 cc intravitreal injections. The vitreous biopsy confirmed VRL which was CD20+, MYD88 L265P PCR+ and ki-67 markedly positive.

He continued to receive alternating rituximab 1 mg/0.1 cc and methotrexate 400 mcg/0.1 cc intravitreal injections in both eyes weekly for a total of 8 injections and underwent systemic stem cell transplantation. Six months post-operatively, he was doing well with a corrected visual acuity of 20/50- right eye and 20/40- left eye. Anterior chamber and anterior vitreous showed faint cells with a clear view of the fundus with no haze. Mild nuclear sclerotic (NS) changes were noted in both eyes, with mild posterior subcapsular (PSC) changes in the right eye.

**Fig. 1:**
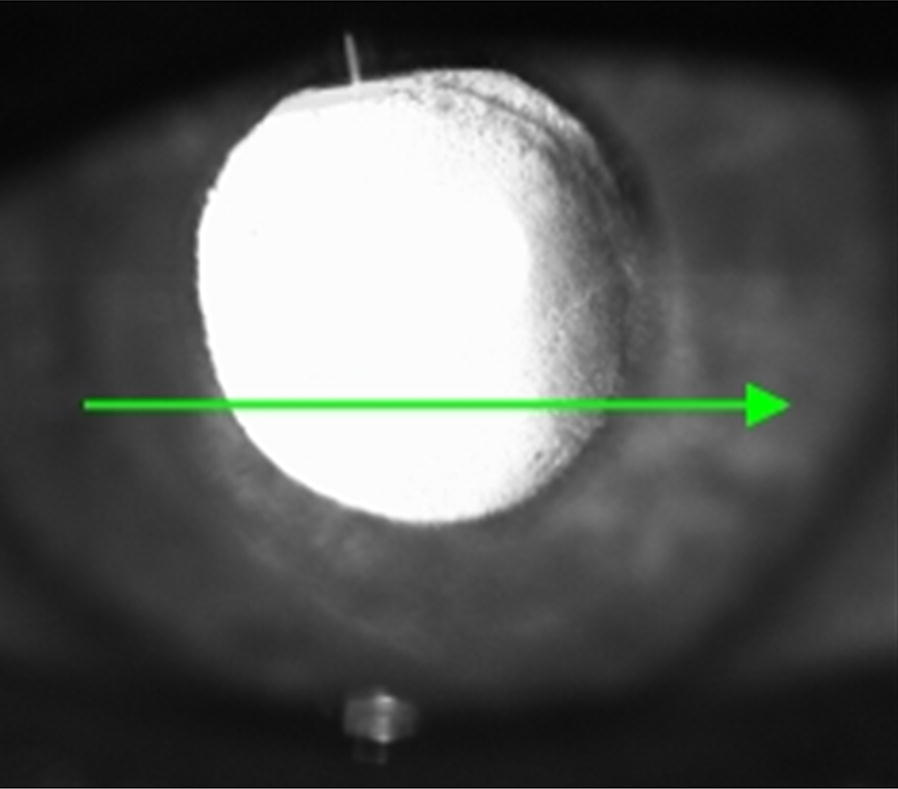
Preop infrared photograph of the right eye shows dense opacification of the vitreous

**Fig. 2:**
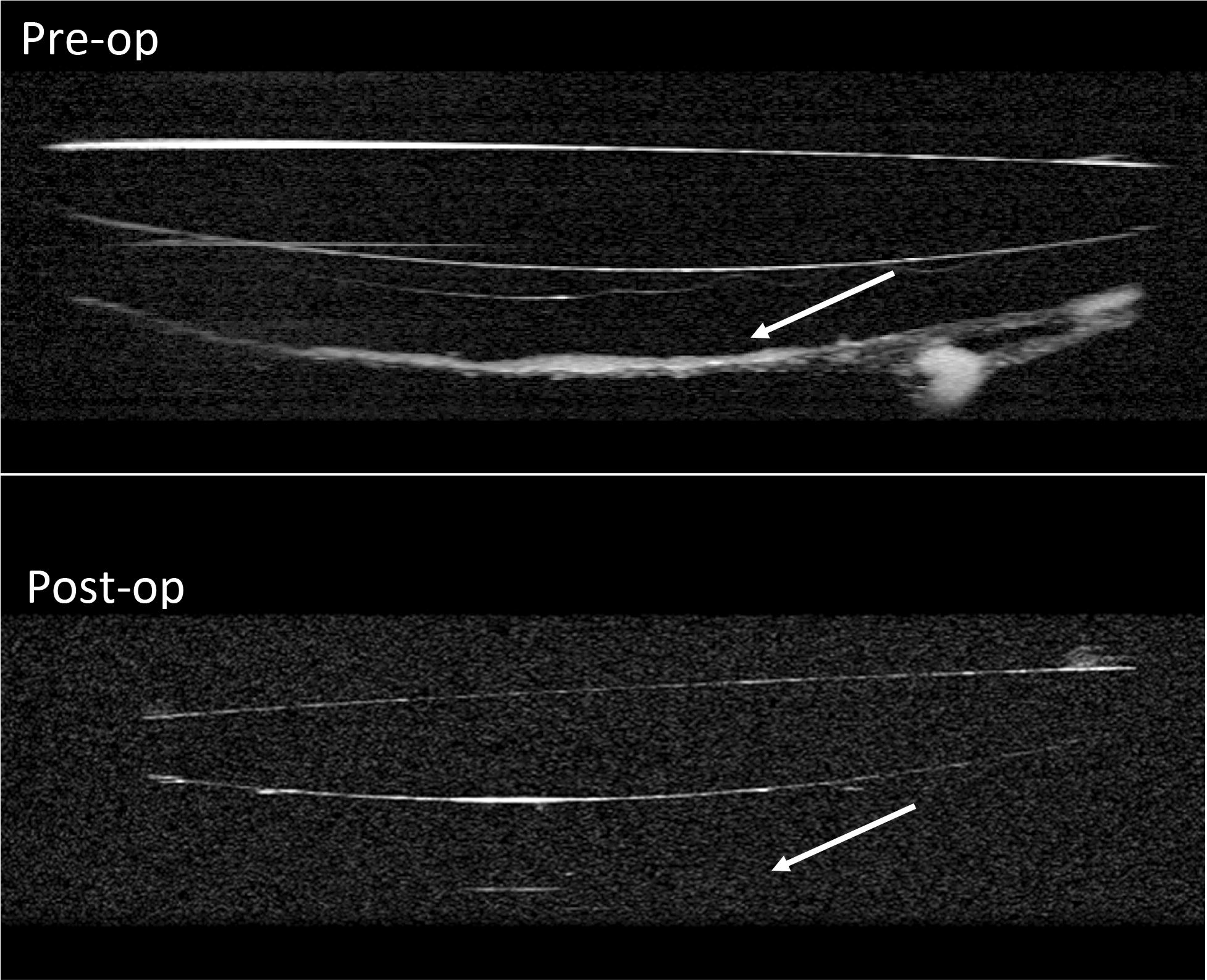
Pre-op and post-op anterior segment OCT of the right eye. Pre-op scan shows formed vitreous with debris, which was later biopsy proven for VRL. Post-operative scan shows complete resolution of the region

Ten months post-operatively, he returned with a 6-week history of blurring of vision worse in the right than in the left eye. His acuity measured counting fingers in the right eye and 20/30 in the left eye, with a dense posterior subcapsular cataract in addition to mild nuclear sclerosis. He had 1+ cells in the anterior chamber and 4+ haze of the vitreous. Given his drop in visual acuity with the progression of cataract in the setting of recurrence of vitreous haze, phacoemulsification was scheduled to get a better view for fundus evaluation. One week later, patient underwent uneventful phacoemulsification with posterior chamber intraocular lens implantation in the right eye, and an aqueous sample was obtained to rule out VRL recurrence. His post-operative visual acuity was 20/40 and a significant haze remained in the right eye. Examination revealed persistent anterior vitreous opacification and cells posterior to the IOL which were well-demonstrated on ASOCT (Figs. 1, 2).

**Fig. 3:**
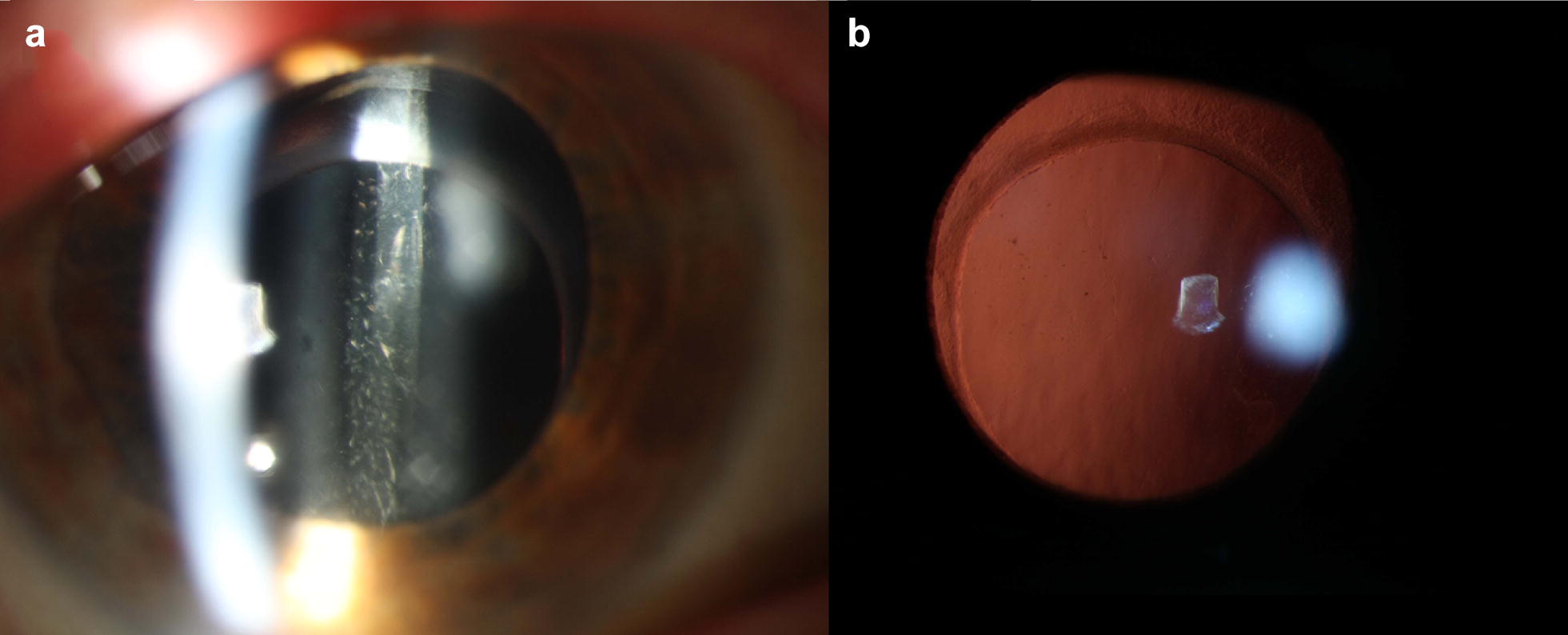
Post-op **a** anterior segment photo and **b** red reflex photo of the right eye. Photos show resolution of the anterior vitreous debris following anterior vitrectomy

**Fig. 4:**
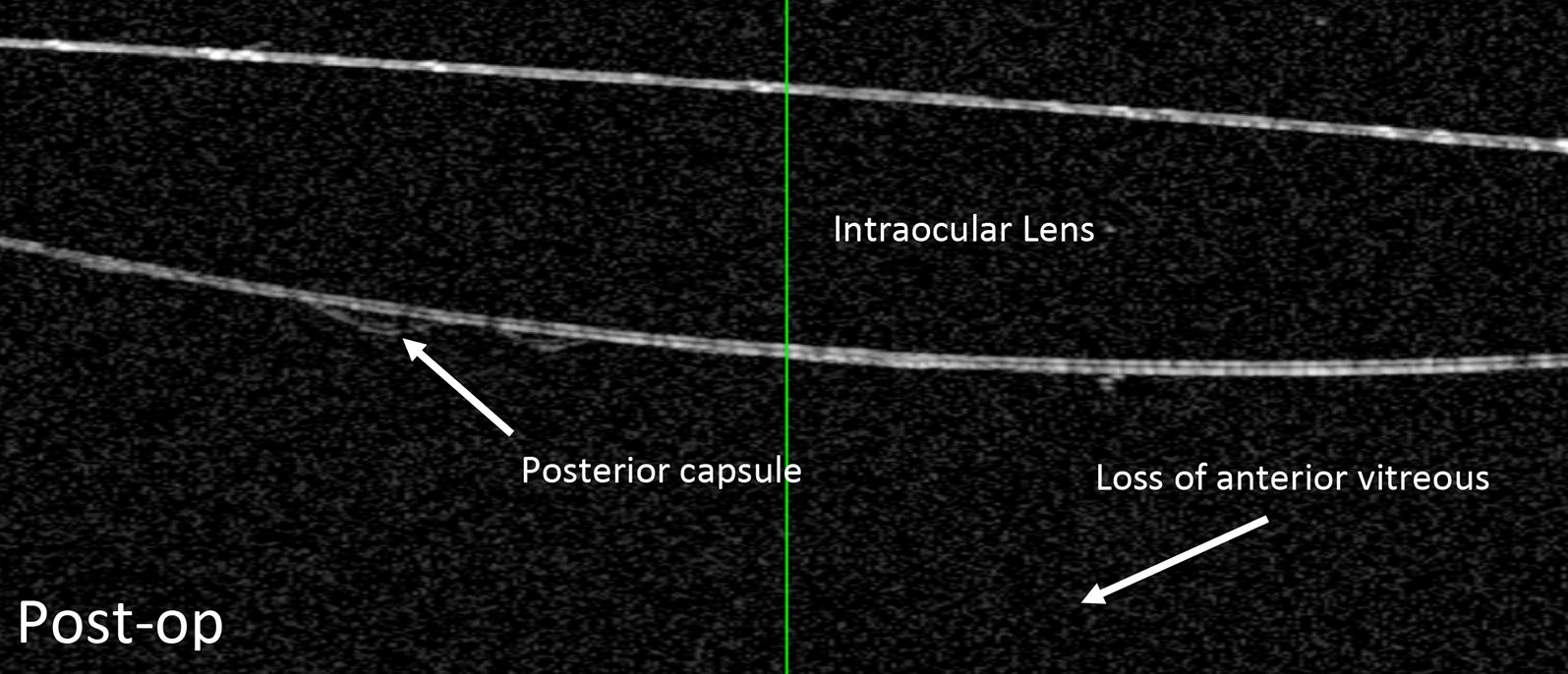
Anterior segment OCT taken after second vitrectomy shows clearance of the anterior vitreous and a well visualized posterior capsule

He was again scheduled for right pars plana vitrectomy, vitreous biopsy and intravitreal methotrexate injection. The goal was to clear the anterior vitreous which had remained behind the natural lens at the time of the initial vitrectomy. Subsequent biopsy results confirmed that the cells were positive for CD20, MYD88 L265P alteration and Ki-67 immunostaining (which showed high proliferation rate > 90%). Three weeks after his second vitrectomy, his vision was 20/100 with 2+ vitreous cell and mild haze, and the ASOCT of the anterior segment showed that the posterior capsule was still present and that there was resolution of the anterior vitreous cells and opacification (Figs. 3, 4). Four months post-operatively, his visual acuity improved to 20/40 + 1 with a mild epiretinal membrane on macula OCT.

## Discussion and conclusions

This case shows that VRL can recur in the formed anterior vitreous of a previously vitrectomized phakic eye. Recurrence has been well-documented in the literature, with moderate vitritis and vitreous haze being reported as most common clinical findings [10, 26, 35]. Hussain et al. [36] published a report of recurrence of VRL in a pseudophakic patient who had previous pars plana vitrectomy. Their conclusion was that the visible cells and opacification were located on the posterior capsular bag. However there is no published ASOCT of the patients. Recurrence has also been described with anterior chamber cells and pseudo-hypopyon in a pseudophakic patient, but no ASOCT findings are described [37]. Saito et al. [38] reviewed 26 eyes with OCT findings of PVRL, however they also did not look at anterior segment OCT.

A case report by Venkatesh et al. [33] described a patient who had a recurrence of VRL. They did not comment on the phakic status of the patient, but they noted that in the previously vitrectomized eye, there were no retro-lenticular cells, while the fellow eye had visible cells. There is no ASOCT published with their findings.

Our report documents a novel finding in recurrence of VRL. Despite the history of vitrectomy, there remained enough anterior vitreous in the retro-lenticular space. Even with thorough 25-gauge or 27-gauge techniques, the space just behind the crystalline lens is difficult to clear without risking injuring to the lens and subsequent cataract formation. This vitreous “pillow” [39] has been described in the literature previously, and although it may have beneficial effects in most patients, it appears to be deleterious in the setting of VRL. In this particular case, it was useful to delineate VRL recurrence of the posterior capsule versus that of the anterior vitreous. Since we could show the cells were present in the anterior vitreous, a second vitrectomy would be useful in clearing the cells.

We did not evaluate the use of ultrasound biomicroscopy (UBM) for imaging the anterior vitreous VRL recurrence. It could be that the anterior vitreous could be imaged similarly with UBM as with ASOCT. Furthermore, it would be expected that UBM may provide additional information of the posterior chamber structures and involvement of the ciliary body and sulcus.

In pseudophakic patients undergoing vitrectomy for VRL, further discussion is warranted as to whether to remove the posterior capsule or not. Although it has benefits of IOL centration and acts as a barrier between the anterior and posterior segment, no study has looked at whether it may act as a scaffold in a similar fashion as does vitreous for VRL recurrence.

Given the course of our patient, we propose that in the setting of biopsy-proven VRL, phakic patients who undergo therapeutic vitrectomy, should also be strongly considered for lens extraction and anterior vitrectomy. In the case of recurrent disease, this particular approach may be beneficial as it may decrease the subsequent need for vitrectomy, and limit recurrences. Also, pseudophakic and previously vitrectomized eyes presenting with VRL should be assessed with ASOCT and there should be strong consideration about removal of the anterior vitreous, should there be any need for surgical intervention. ASOCT allows determination as to whether cells have collected in the anterior vitreous or on the posterior capsule. This allows for appropriate medical and surgical management to be undertaken for recurrence of VRL.

## Data Availability

Not applicable.

## Abbreviations

ASOCT: anterior segment optical coherence tomography
CF: count fingers
CNS: central nervous system
DLBCL: diffuse large B cell lymphoma
IOP: intraocular pressure
MRI: magnetic resonance imaging
NS: nuclear sclerotic
OCT: optical coherence tomography
PCR: polymerase chain reaction
PSC: posterior subcapsular cataract
UBM: ultrasound biomicroscopy
VRL: vitreoretinal lymphoma
